# Mucinous cystadenoma of the appendix misdiagnosed as cystic hydatid disease of the liver: a case report

**DOI:** 10.1186/1752-1947-2-218

**Published:** 2008-06-25

**Authors:** Andreas Krieg, Jan Schulte am Esch, Ludger W Poll, Stefan Braunstein, Wolfram T Knoefel

**Affiliations:** 1 Department of General and Visceral Surgery, Heinrich Heine-University, Duesseldorf, Germany; 2 Institute of Diagnostic Radiology, Heinrich Heine-University, Duesseldorf, Germany; 3 Institute of Pathology, Heinrich Heine-University, Duesseldorf, Germany

## Abstract

**Introduction:**

Primary neoplastic lesions presenting with a mucocele of the appendix are very rare and can be divided into benign variants of mucinous adenomas or cystadenomas, mucinous tumours of uncertain malignant potential or mucinous cystadenocarcinomas. Most of these tumourous mucoceles are asymptomatic and are found incidentally. The major complication of neoplastic mucinous appendiceal tumours is the development of a pseudomyxoma peritonei due to spreading of mucin-producing cells within the abdominal cavity.

**Case presentation:**

A 44-year-old man presented with a history of non-specific symptoms of right upper abdominal pain. Abdominal ultrasound and computed tomography scan identified a cystic mass consistent with the morphological characteristics of an echinococcal hydatid cyst. After completing systemic albendazole therapy, an explorative laparotomy revealed a cystic tumour of the appendix. Ileocaecal resection was performed and pathology reports confirmed the diagnosis of a mucinous cystadenoma of the appendix. The postoperative course was uneventful.

**Conclusion:**

Here we present the case of a man with a mucinous cystadenoma of the appendix mimicking cystic hydatid disease. We discuss the importance of re-evaluation and differential diagnostic reflections in cases of appendiceal mucocele.

## Introduction

Mucocele of the appendix is a rare cystic dilatation of the appendiceal lumen caused by mucinous secretions and consecutive retention of mucus. It can be caused by either non-neoplastic or primary epithelial neoplastic disease [1,2]. The neoplastic variants are caused by mucus-producing epithelial neoplasms including mucinous adenomas or cystadenomas, mucinous tumours of uncertain malignancy and mucinous cystadenocarcinomas [2-5]. Most of these tumours are asymptomatic and may be found incidentally [1,2,5-7]. Others become symptomatic because of inflammation in, for example, acute appendicitis or as a cause of non-specific abdominal pain [5]. A major complication of neoplastic mucinous appendiceal tumours is the development of pseudomyxoma peritonei, which has a high rate of morbidity and mortality [8].

Here we report a case of a man with a mucocele caused by a mucinous cystadenoma mimicking cystic hydatid disease and discuss the importance of differential diagnostic reflections in cases of appendiceal mucocele.

## Case presentation

**Figure 1:**
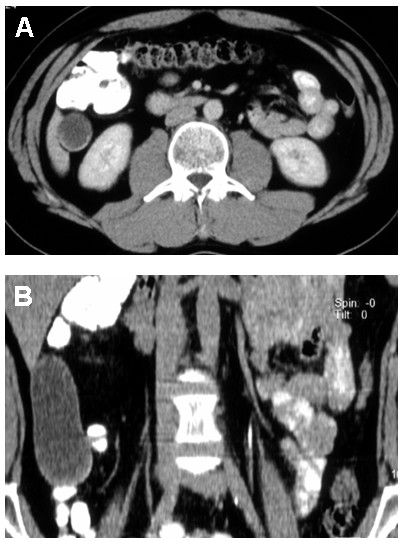
**Contrast-enhanced computed tomography of the abdomen reveals a well-delineated mucinous mass with curvilinear calcification**. **A) **The axial view indicates that the mass is located at the inferior tip of the right liver lobe. The surrounding fat tissue does not show any fat-stranding. **B) **The coronal view clearly shows the cranio-caudal extension of the mass from the liver segment six to the ileocaecal area.

A 44-year-old man presented with a history of non-specific symptoms of right upper abdominal pain for 6 years. Abdominal ultrasound (US) revealed a cystic mass containing low level internal echoes and sonic shadowing. Computed tomography (CT) revealed, in the axial and coronal views, a cystic mass with curvilinear calcifications extending from segment six of the right hepatic lobe into the right lower quadrant, consistent with the morphological characteristics of an echinococcal hydatid cyst (Figure 1A and 1B). The patient was employed as a hunt assistant and was exposed to dogs and foxes and their faeces. Serodiagnostic results for echinococcal antigens and antibodies were within normal limits. A surgical resection of the cyst by pericystectomy was planned and systemic albendazole therapy was started prior to cyst evacuation. Four weeks after systemic albendazole therapy, an exploratory laparotomy via a right subcostal incision with extension in the midline was performed.

**Figure 2:**
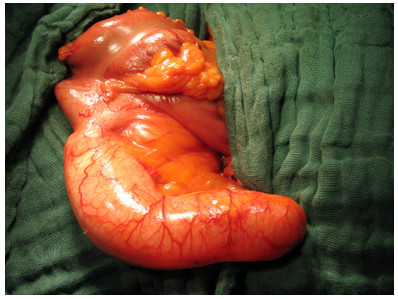
**Intraoperatively identified appendiceal mucocele**. The mucocele was in the antecaecal location and on macroscopic assessment showed a smooth serosal surface without peritoneal implants.

**Figure 3:**
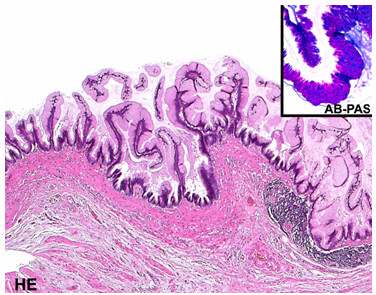
**Histological characteristics of the mucinous cystadenoma**. Haematoxylin and eosin stain shows a villous growth pattern and positive staining with periodic acid Schiff.

During exploration of the abdominal cavity the cystic mass, which measured 17.5 cm in length and 4.5 cm in diameter, was identified as a cystic antecaecal appendix with a smooth serosal surface involving the base of the appendix (Figure 2). Due to the involvement of the appendiceal base and the risk of perforation, we decided against a classical appendectomy, which is recommended in uncomplicated mucocele of the appendix, and performed an ileocaecal resection with ileoascendostomy. Intra-operatively, no peritoneal tumour implants or ascites were conspicuous. Pathological examination revealed a mucinous cystadenoma with a partly flat and villous growth pattern, staining positively with periodic acid Schiff. The nuclei were hyperchromatic with low-grade atypia and single mitotic figures. Dystrophic calcifications were seen in areas with epithelial denudation and extended extravasated mucin (Figure 3). The patient's postoperative course was uneventful and annual colonoscopy and ultrasound as follow-up were recommended because of the potential of metachronic cancer and the development of pseudomyxoma peritonei.

## Discussion

The incidence of mucoceles of the appendix is reported to be 0.2% to 0.3% [2]. The non-neoplastic variety is caused by an obstruction close to the base of the appendix, usually by a faecalith, resulting in the accumulation of mucus and subsequent dilatation. The neoplastic variant is caused by a mucinous epithelial neoplasm, such as benign neoplasms, including mucinous adenomas or cystadenomas, mucinous tumour of uncertain malignant potential or the malignant variant of mucinous cystadenocarcinoma [2-5]. Approximately 25% to 50% of mucoceles are asymptomatic and are found incidentally at physical examination or during abdominal imaging or surgery [1,2,5-7]. Other clinical manifestations include symptoms caused by acute appendicitis or non-specific abdominal pain [5]. In particular, malignant mucinous cystadenocarcinoma may be symptomatic due to invasion of adjacent organs. One major complication of neoplastic mucinous appendiceal tumours is the development of pseudomyxoma peritonei due to neoplastic mucus-producing cells within the abdominal cavity [8]. These patients may become symptomatic because of abdominal pain, distension and palpable masses in the abdomen as well as nausea, vomiting or fatigue. Recurrent disease often involves the bowel surface and is associated with intense fibrosis leading to adhesions and intestinal obstruction, which often is the cause of death. The five-year survival in patients with pseudomyxoma peritonei has been reported to be 53% [8]. As there is a reported co-incidence of mucinous appendiceal tumours and colonic neoplasms, colonoscopy should, if possible, be performed before surgical treatment as well as during follow-up [4]. Diagnostic clues of appendiceal mucocele at abdominal US are a round-, ovoid-, sausage-, pear- or chicken drumstick-shaped cystic mass with a variable intraluminal echotexture that in most cases shows low-level internal echoes or septae. A strong echo with posterior sonic shadowing, caused by dystrophic curvilinear calcifications of the appendiceal wall as a result of chronic inflammation, might be obvious as was seen in the case presented here [6,7,9].

Cross sectional imaging such as CT is superior to US in the evaluation of mucoceles of the appendix because it demonstrates the topographic anatomical relationship between the caecum and the mucocele. In addition, CT is more sensitive than US in the detection of mural calcifications within the mucinous neoplasm. The appearance at CT scanning is characterized by a well-encapsulated cystic structure with either an enhancing smooth thin or thick wall, with or without mural calcification. The detection of mural curvilinear calcifications is highly suggestive of the diagnosis, but is detectable in less than 50% of cases [6]. Kim et al. reported the appearance of small enhancing nodules in the wall of mucinous cystadenocarcinomas, which might enable differentiation between benign and malignant mucoceles [9].

Several diseases including hydrosalpinx, ovarian cysts or renal cysts have been reported as mimicking appendiceal mucocele in their US and CT appearance as well as in their uncharacteristic clinical symptoms [10,11]. To our knowledge there are no reports of mucinous appendiceal neoplasms presenting as cystic hydatid disease of the liver.

Cystic hydatid disease, caused by E*chinococcus granulosus*, has an uneven geographical distribution in Europe, presenting with well-defined single or multiple cysts on CT scan that may be uniloculated or multiloculated and either thin or thick walled. Calcification, daughter cysts and germinal membrane detachment may also be present. Usually no rim enhancement is evident unless the hydatid cyst is superinfected [12]. Cystic hydatid disease can occur anywhere but is predominantly found in the right lobe of the liver and may be characterized by nonspecific symptoms [13].

Although serodiagnosis was negative, in our case the morphological appearance on CT, the localization with topographic relation to the right liver lobe and the medical history made the diagnosis of a cystic hydatid disease most likely. It is worth noting that the sensitivity and specificity of serodiagnostic tests for Echinococcosis have been reported to range between 80% and 90% and therefore results might be negative despite hydatid disease being present [14].

New therapeutic approaches in the treatment of hydatid disease include percutaneous punction of the cystic lesions followed by aspiration of the internal fluid and injection of alcohol containing solutions, and re-aspiration. This so-called PAIR procedure should be repeated several times [15]. In this reported case, as we were unable to rule out the existence of a mucinous cystadenoma, the PAIR procedure seemed inappropriate, as it might easily have led to the feared major complications of pseudomyxoma peritonei by translocating mucus-producing neoplastic cells within the abdominal cavity along the trajectory of canula.

## Conclusion

The case presented shows the importance of re-evaluation and differential diagnostic reflections in cases of the very rare entity of mucocele of the appendix. In particular, in cases with high suspicion for hydatid disease but with negative serodiagnosis, surgical exploration is recommended rather than the interventionally performed PAIR procedure.

## Abbreviations

CT: computed tomography; PAIR: puncture aspiration injection re-aspiration; US: ultrasound.

## Competing interests

The authors declare that they have no competing interests.

## Consent

Written informed consent was obtained from the patient for publication of this case report and accompanying images. A copy of the written consent is available for review by the Editor-in-Chief of this journal.

## Authors' contributions

AK and JSAE performed the surgery, conducted the acquisition, analysis and interpretation of data and drafted the manuscript, LWP performed the analysis and interpretation of the computed tomography scans, SB performed the pathological analysis, WTK made substantial contributions to the conception, acquisition and interpretation of data and revised the manuscript critically. All authors read and approved the final manuscript.
